# The effectiveness of different hormone protocols for improving ovarian function before ovum pick-up in crossbred Japanese black cattle

**DOI:** 10.14202/vetworld.2024.1362-1369

**Published:** 2024-06-21

**Authors:** Jatuporn Kajaysri, Apiradee Intrarapuk

**Affiliations:** Department of Science, Technology and Innovation, Faculty of Science, Chulabhorn Royal Academy, Bangkok, Thailand

**Keywords:** estradiol benzoate, follicle-stimulating hormone, gonadotropin hormone-releasing hormone, Japanese black cow, ovum pick-up

## Abstract

**Background and Aim:**

Producing and transferring embryos *in vitro* are profitable for enhancing premium beef genetics. Reducing costs and enhancing the effectiveness of hormone protocols before ovum pick-up (OPU) yield advantages. This study aimed to confirm that estradiol benzoate (EB) treatment resulted in more medium- and large-sized follicles before OPU and of higher oocyte quality after OPU than non-hormonal treatments, comparable to those undergoing gonadotropin-releasing hormone (GnRH) with follicle-stimulating hormone (FSH) plus prostaglandin F_2α_ (PGF_2α_) and progesterone-releasing controlled internal drug release (CIDR) or EB with progesterone-releasing CIDR hormonal treatments.

**Materials and Methods:**

30 crossbred Japanese black cows were divided into five equal groups, which were either untreated or treated with different hormone protocols before OPU. Group 1 (cows in estrus) and group 2 (cows in diestrus) were the untreated controls. Cows in group 3 were treated with GnRH + FSH + CIDR + PGF_2α_. Cows in group 4 received EB, and those in group 5 received EB + CIDR + PGF_2α_. After administering the protocols, all cow follicles were examined through ultrasonography and categorized by size. Subsequently, all cows underwent OPU, and the oocytes were collected and graded from A to D according to standard criteria.

**Results:**

Group 3 presented the highest large follicle numbers, and groups 3–5 had more medium follicle numbers, not different among groups but they had this parameter more than those of control groups 1 and 2. Moreover, groups 3–5 did not differ in combined grades A + B oocytes (good-quality oocytes). Groups 3 and 4 had more A + B oocytes than control groups 1 and 2, whereas group 5 was not different in this parameter from group 1.

**Conclusion:**

Among the three hormone protocols, EB treatment proved the most cost-effective and efficient, yielding more high-quality oocytes compared to the non-treatment protocols. To reduce the limitations of EB use in the future, this study suggests researching natural EB phytoestrogens as alternative treatments for improving ovarian function before OPU in other cattle breeds.

## Introduction

*In vitr*o fertilization (IVF) and *in vitr*o maturation (IVM) for embryo production and embryo transfer can boost the genetics of living cattle and enhance the livestock industry through increased herd productivity in beef and dairy cattle [1–4]. Embryo transfer in cattle costs twice as much as artificial insemination. In addition, if practitioners prefer to use embryo transfer as a biotechnological technique rather than artificial insemination, they must produce cheaper but viable embryos [2]. Previously, embryo transfer using *in vivo* superovulation-fertilized bovine embryos was the standard procedure [5–7]. Recently, *in vitro* embryo production has expanded commercially through the use of embryo transfer. This results in increased calf production [8–10]. *In vitro* production (IVP) still involves the removal of oocytes using ultrasound-guided ovum pick-up (OPU) for follicle puncture [1, 11–14]. OPU is currently used and is important for enhancing the efficiency of IVF in Japanese black cattle [11]. OPU technology can collect high-quality oocytes from live cattle [4, 15, 16]. This technique allows for the collection of oocytes at any point during the estrus cycle, including early pregnancy stages. Oocytes accumulate in the ovaries of juvenile calves, prepubertal heifers, and cows with reproductive issues [17–20]. The accumulated oocytes can be fertilized *in vitro* with sperm from different bulls, thus increasing the genetic variation in embryo production [21]. By accelerating the elimination of less desirable genotypes and promoting the survival of preferred ones, OPU-IVP enhances cattle genetics in a shorter timeframe [22]. In the cattle industry, the demand for OPU-IVP embryos is projected to rise [21, 23, 24].

In addition, gonadotropin hormone-releasing hormone (GnRH), follicle-stimulating hormone (FSH), prostaglandin F_2α_ (PGF_2α_), and intravaginal progesterone-releasing hormone treatments can increase the efficiency of OPU-IVF followed by IVP embryo production. This is a standard protocol for collecting oocytes that have matured *in vivo* through OPU in Japanese black cattle [24]. GnRH and FSH treatments super-stimulate mature oocytes, which are then used to create high-quality embryos *in vitro* [24].

Exogenous estradiol benzoate (EB) administration at the luteal phase before OPU increases the number of medium-sized follicles and good-quality oocytes after OPU collection in Japanese black cattle compared with no EB treatment at the same phase [2]. Regardless of the developmental stage of the dominant follicles at exogenous EB administration, EB suppressed the dominant follicle, which consistently led to the formation of a new follicular wave on an average of 4.3 days later [25, 26]. Moreover, using EB to coordinate ovulatory follicle growth during proestrus enables manipulation of the dynamics of ovarian follicular wave development [27, 28]. Nevertheless, little information exists for comparing non-EB treatments during the follicular phase with EB treatments during the luteal phase.

Previous studies by Hidaka *et al*. [2] and Kaminski *et al*. [29] suggest that administering progesterone and EB can synchronize and regulate follicular wave development. The OPU without prior hormonal treatment can be performed and repeated in 1–3 weeks [30]. However, this protocol is able to collect fewer good-quality oocytes, compared with OPU with prior EB treatment [2]. In beef cattle, there is scant research on the relative efficiencies of EB and EB-progesterone combination treatment in terms of follicle and good-quality oocyte numbers before OPU.

This study proposed that EB administration during the follicular and luteal phases would lead to a larger number of medium- and large-sized follicles before OPU and better-quality oocytes after OPU in comparison to non-hormonal treatments. Our hypothesis assumed that these parameters would mirror those of hormonal therapies incorporating GnRH, FSH with PGF_2α_, progesterone-releasing controlled internal drug release (CIDR), and EB with progesterone-releasing CIDR.

## Materials and Methods

### Ethical approval

The Animal Ethics Committee of the Faculty of Veterinary Medicine at Mahanakorn University of Technology, Bangkok, Thailand, approved the use of the experimental animals in this study (Approval no. ACUC-MUT-2022-006).

### Study period and location

The study was conducted from March to October 2019 and included follicle determinations, follicle aspirations, and oocyte evaluations on a private beef farm in Kanchanaburi province (western region), Thailand.

### Experimental animals

Thirty primiparous and pluriparous crossbred Japanese black cows were used for OPU. In evaporative cooling-free stall barns, cows aged 3–7 years and weighed between 250 and 450 kg were housed (close system). Cows were given daily rations of 14.00% crude protein concentrates and grass silage, along with unlimited access to clean water. During the experiment, the animals lived and moved freely in the stall barns. The temperature and humidity inside varied between 26°C and 29°C and 70% and 80%, respectively. All the cows’ reproductive systems were functioning normally, free from anatomical disorders. According to the Department of Livestock Development’s recommendations, the cows were dewormed and immunized annually.

### Experimental design

**Figure-1:**
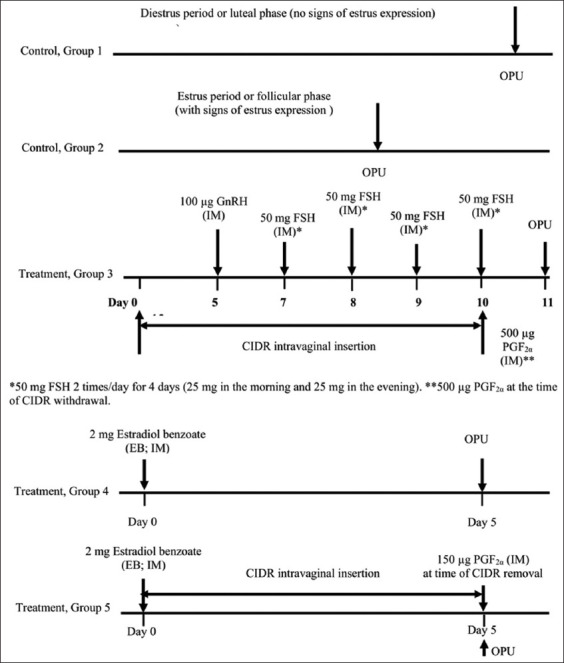
Hormone protocols for enhancing ovarian function before ovum pick-up in crossbred Japanese black cattle. IM=Intramuscular.

Thirty cows were randomly assigned to five groups of six cows each. The control groups were group 1 (cows in estrus, i.e., follicular phase, with estrus expression) and group 2 (cows in diestrus, i.e., luteal phase, without estrus signs). These groups received no hormones before OPU. Group 3 was treated with 100 μg GnRH (Receptal^®^; MSD Animal Health Co., Ltd, Wellington, New Zealand), 200 mg FSH (divided into 8 injections at 12-h intervals) twice daily for 4 days (Folltropin^®^; Bioniche Animal Health Co., Ltd, Ontario, Canada), 1.9 g P_4_ hormone impregnated intravaginally (CIDR; Eazi-Breed^®^, Pfizer Animal Health Co., Ltd, Hamilton, New Zealand) for 10 days, and 500 μg PGF_2α_ (cloprostenol; Estrumate^®^; MSD Animal Health Co.) at the time of CIDR withdrawal. Group 4 (cows in the luteal phase) received 2 mg EB (Lamthong Karnpat Pharmaceutical Factory Co., Ltd, Bangkok, Thailand), with OPU on day 5. Group 5 (cows in the luteal phase) received 2 mg EB and 150 μg PGF_2α_ + CIDR for 5 days, with OPU being performed on the day of CIDR removal. Figure-1 describes the experimental design of the hormone protocols and OPU sessions for all study groups. The hormone treatment protocol for group 3 was modified from that of Egashira *et al*. [24]; our study adjusted the quantities of GnRH and FSH injections, and Nilchuen *et al*. [31], administered the CIDR in the protocol. Group 4 was modified from that of Hidaka *et al*. [2]; our study changed the OPU hours after EB treatment. Group 5 was modified from that of Cavalieri *et al*. [1]; our study changed the day of PGF_2α_ treatment.

### Follicle determination

Cow follicular sizes were determined through ultrasonography using a 7.5-MHz linear transducer scanner (Sonoscape A6^®^, SonoScape Co., Ltd., Shenzhen, China) after the hormone protocols were administered. Following ultrasonography, we categorized ovarian follicle sizes for all cows into small (<5 mm), medium (5–9 mm), or large (>9 mm) based on method described by Egashira *et al*. [24] with some modification; Our study amended the medium and large follicle sizes to be larger than those of the previous study [24].

### Follicle aspirations and OPUs

After determining the follicular sizes through ultrasonography (Sonoscape A6^®^), the follicles were punctured for OPU by the same veterinarian using a 17-gauge disposable single-lumen cow ovum-vacuuming needle (COVA Needle, Watanabe Tecnologia Aplicada, São Paulo, Brazil). The needle was outfitted with a 7.5-MHz micro-convex array transducer for guided real-time ultrasound (Sonoscape A6^®^), and the caudal portion of the needle was connected to a 50-mL centrifuge tube using an aspiration tubing set. The aspirator (Bovine Follicular Aspiration Pump [20032], Watanabe Tecnologia Aplicada, São Paulo, Brazil) with a 20 mL/min aspiration rate and 115 mmHg vacuum pressure was equipped with a needle, centrifuge tube, and aspiration tubing for follicular puncture. The cumulus-oocyte complexes (COCs) and follicular fluid from follicular aspiration were collected in a collection medium in 50-mL centrifuge tubes and stored at 35°C in a warm water bath. The collection media contained Lactate Ringer’s Solution (LRI, GHP, Pathum Thani, Thailand), 1 mg/mL polyvinylpyrrolidone (PVP40; Sigma-Aldrich, St. Louis, MO, USA), 20 IU/mL heparin (Heparin LEO, LEO Pharma, Bangkok, Thailand), and 0.1 mg/mL penicillin-streptomycin (Pen-Strep, Gibthai, Bangkok, Thailand). During follicular aspiration, all cows were injected with a single dose of 100 mg (5 mL) of 2% lidocaine hydrochloride (Lidocaine 2% Injection, Union Drug Laboratories, Bangkok, Thailand) to relax the rectum and reduce abdominal tension, which was required to palpate the ovaries.

### Oocyte evaluations

The oocytes were collected through OPU and evaluated under a 10× stereoscope (Nikon SMZ800N, Nikon, Tokyo, Japan) according to the morphological criteria described by Hidaka *et al*. [2] and Kouamo *et al*. [32]. Grade-A COCs had uniform ooplasm and >4 layers of compressed cumulus cells. Grade-B cumulus cells were compacted and arranged in 3–4 layers, each with uniform ooplasm. Grade-C cumulus cell layers were less compact and contained uneven ooplasm with black granules. Grade-D oocytes were denuded and lacked cumulus cells. Grade E oocytes had enlarged cumuli with jelly-like structures. In this study, grade E oocytes were adjusted and not calculated for statistical analyses. The collected oocytes in each group were of cumulus quality and categorized into four grades of A–D.

### Statistical analysis

All statistical analyses were performed using GraphPad Prism software, Version 5.0 (GraphPad Software, Inc., San Diego, CA, USA) or Statistical Package for the Social Sciences, Version 26.0 (IBM, Armonk, NY, USA). Tables-1 and 2 show the descriptive statistics for all variables and are presented as frequencies. Tables-3–6 are presented as means ± standard deviation (SD). The Kolmogorov–Smirnov test was used to check for normally distributed data. Nonparametric testing was used to compare different groups when applicable. Follicle and oocyte numbers were compared between groups using the Mann–Whitney U test, and follicle size and oocyte quality between groups were evaluated using Wilcoxon’s signed rank test. For all tests, p < 0.05 was considered statistically significant.

**Table-1:** Numbers and percentages of small, medium, and large follicles after administering hormone protocols in cows.

Group	Number of cows per group (n)	Follicle size			Total (%)

		Small (<5 mm) (%)	Medium (5–9 mm) (%)	Large (>9 mm) (%)	
1*	6	155 (92.26)	12 (7.14)	1 (0.60)	168 (100)#
2**	6	127 (86.99)	13 (8.90)	6 (4.11)	146 (100)#
3	6	11 (8.73)	50 (39.68)	65 (51.59)	126 (100)#
4	6	98 (68.06)	46 (31.94)	0 (0)	144 (100)#
5	6	105 (74.47)	36 (25.53)	0 (0)	141 (100)#
Total	30	496 (68.41)	157 (21.66)	72 (9.93)	725 (100)

* Control group 1: Cows in diestrus without signs of estrus expression.

** Control group 2: Cows in estrus showing signs of estrus expression.

# Sum equals 100% in the same row

**Table-2:** Numbers and percentages of graded oocytes in each group in grades A, B, C, and D.

Group	Number of cows per group (n)	Oocyte quality				Total (%)

		Grade A (%)	Grade B (%)	Grade C (%)	Grade D (%)	
1*	6	6 (7.23)	25 (30.12)	17 (20.48)	35 (42.17)	83 (100)#
2**	6	3 (4.00)	21 (28.00)	11 (14.67)	40 (53.33)	75 (100)#
3	6	30 (44.78)	16 (23.88)	6 (8.95)	15 (22.39)	67 (100)#
4	6	14 (18.92)	34 (45.95)	8 (10.81)	18 (24.32)	74 (100)#
5	6	18 (26.09)	24 (34.78)	6 (8.70)	21 (30.43)	69 (100)#
Total	30	71 (19.29)	120 (32.62)	48 (13.04)	129 (35.05)	368 (100)

* Control group 1: Cows in diestrus without signs of estrus expression.

** Control group 2: Cows in estrus showing signs of estrus expression.

# Sum equals 100% in the same row

**Table-3:** Mean ± SD of the numbers of small, medium, and large follicles in cows within each group after administering hormone protocols.

Group	Number of cows per group (n)	Follicle size		

		Small (<5 mm)	Medium (5–9 mm)	Large (>9 mm)
1	6	25.83 ± 5.31^a^	2.00 ± 1.41^b^	0.17 ± 0.41^c^
2	6	21.17 ± 3.54^a^	2.17 ± 1.17^b^	1.00 ± 0.63^b^
3	6	1.83 ± 0.41^a^	8.33 ± 1.75^b^	10.83 ± 2.04^b^
4	6	16.33 ± 3.67^a^	7.67 ± 2.16^b^	0.00 ± 0.00^c^
5	6	17.50 ± 3.78^a^	6.00 ± 1.67^b^	0.00 ± 0.00^c^

^a,b,c^Significant difference in the same row (p < 0.05). SD=Standard deviation

**Table-4:** Mean ± SD of the numbers of small, medium, and large follicles in cows among groups after administering hormone protocols.

Group	Number of cows per group (n)	Follicle size		

		Small (<5 mm)	Medium (5–9 mm)	Large (>9 mm)
1	6	25.83 ± 5.31^a^	2.00 ± 1.41^a^	0.17 ± 0.41^a^
2	6	21.17 ± 3.54^a,b^	2.17 ± 1.17^a^	1.00 ± 0.63^b^
3	6	1.83 ± 0.41^c^	8.33 ± 1.75^b^	10.83 ± 2.04^c^
4	6	16.33 ± 3.67^b^	7.67 ± 2.16^b^	0.00 ± 0.00^a^
5	6	17.50 ± 3.78^b^	6.00 ± 1.67^b^	0.00 ± 0.00^a^

^a,b,c^Significant difference in the same column (p < 0.05). SD=Standard deviation

**Table-5:** Mean ± SD numbers of graded oocytes in grades A, B, C, and D among groups.

Oocyte grade	Group 1 (n = 6)	Group 2 (n = 6)	Group 3 (n = 6)	Group 4 (n = 6)	Group 5 (n = 6)
Grade A	1.00 ± 0.63^a^	0.50 ± 0.84^a^	5.00 ± 0.89^a^	2.33 ± 0.52^a^	3.00 ± 0.63^a^
Grade B	4.17 ± 1.17^b^	3.50 ± 1.52^b^	2.67 ± 0.82^b^	5.67 ± 0.82^b^	4.00 ± 1.55^a^
Grade C	2.83 ± 1.17^a,b^	1.83 ± 2.56^a,b^	1.00 ± 0.89^c^	1.33 ± 0.52^c^	1.00 ± 0.63^b^
Grade D	5.83 ± 1.47^c^	6.67 ± 2.16^c^	2.50 ± 0.55^b^	3.00 ± 0.63^a^	3.50 ± 0.55^a^

^a,b,c^Significant difference in the same column (p < 0.05). SD=Standard deviation

**Table-6:** Mean ± SD numbers of graded oocytes in grades A, B, C, and D and combined oocyte grades A + B and C + D among groups.

Oocyte grading	Group 1 (n = 6)	Group 2 (n = 6)	Group 3 (n = 6)	Group 4 (n = 6)	Group 5 (n = 6)
Grade A	1.00 ± 0.63^a^	0.50 ± 0.84^a^	5.00 ± 0.89^b^	2.33 ± 0.52^c^	3.00 ± 0.63^c^
Grade B	4.17 ± 1.17^a^	3.50 ± 1.52^a,b^	2.67 ± 0.82^b^	5.67 ± 0.82^c^	4.00 ± 1.55^a,b,c^
Grade C	2.83 ± 1.17^a^	1.83 ± 2.56^a,b^	1.00 ± 0.89^b^	1.33 ± 0.52^b^	1.00 ± 0.63^b^
Grade D	5.83 ± 1.47^a^	6.67 ± 2.16^a^	2.50 ± 0.55^b^	3.00 ± 0.63^b,c^	3.50 ± 0.55^c^
Grade A + B	5.17 ± 0.98^a,b^	4.00 ± 2.00^a^	7.67 ± 1.37^c^	8.00 ± 0.89^c^	7.00 ± 2.10^b,c^
Grade C + D	8.67 ± 1.97^a^	8.50 ± 4.51^a,b^	3.50 ± 1.05^c^	4.33 ± 0.82^c^	4.50 ± 1.05^b,c^

^a,b,c^Significant difference in the same row (p < 0.05). SD=Standard deviation

## Results

Ovarian follicle dynamics following the different hormone protocols were expressed in order of the number of each categorized follicle size in the ovaries of all cows. In total, 725 follicles were detected in all groups, with 168 (23.17%), 146 (20.14%), 126 (17.38%), 144 (19.86%), and 141 (19.45%) in groups 1, 2, 3, 4, and 5, respectively. Among the cows in groups 1, 2, 4, and 5, the numbers and percentages of small follicles were the highest compared with the numbers and percentages of other follicular sizes. Conversely, the number and percentages of small follicles in group 3 were lower than those of medium and large follicles in this group. Table-1 presents the numbers and percentages of follicles for all groups, categorized as small, medium, and large.

Group 3 had the fewest small follicles, whereas among the other four groups, the number of small follicles was highest (p < 0.05) compared with the numbers of other follicular sizes within the same groups. Table-3 compares the means ± SD of small, medium, and large follicles within each group. Group 3 had the largest and fewest small follicles compared with the remaining four groups (p < 0.05). Moreover, the untreated control groups (groups 1 and 2) presented fewer medium follicles than groups 3–5 (p < 0.05) and tended to have more small follicles than those of the hormone-treated groups. Table-4 compares the means ± SD of the small, medium, and large follicles among all groups.

Oocytes were collected from various size follicles in each group through OPU. In total, 368 oocytes (without grade E oocytes) were collected from all groups following OPU from 725 follicles (including all follicular sizes in all groups), yielding a 50.76% oocyte recovery rate. Of the 368 collected oocytes, 83 (22.55%), 75 (20.38%), 67 (18.21%), 74 (20.11%), and 69 (18.75%) were from groups 1, 2, 3, 4, and 5, respectively. The collected oocytes in each group were evaluated for cumulus quality (grades A–D). The cows in groups 1 and 2 had the highest numbers and percentages of D-grade oocytes. Conversely, the cows in group 3 displayed the highest number and percentage of grade-A oocytes, whereas the cows in groups 4 and 5 presented the highest numbers and percentages of grade-B oocytes. Table-2 shows the number and percentage of graded oocytes in each group.

Table-5 presents the mean numbers (means ± SD) of oocytes from grades A–D in each group. Groups 1 and 2 had the most grade-D oocytes; group 3 had the most grade-A oocytes, and group 4 had the most grade-B oocytes (p < 0.05). The mean numbers of grades A, B, and D oocytes did not significantly differ for group 5, which had the fewest grade-C oocytes (p < 0.05).

Combining groups A + B and C + D showed the most grade-A oocytes in group 3 (p < 0.05), and groups 4 and 5 had significantly more grade-A oocytes than groups 1 and 2 (p < 0.05). Group 4 had the most grade-B oocytes with quantities greater than those of groups 1, 2, and 3 (p < 0.05), but they did not significantly differ from those of group 5. Conversely, the results revealed that the mean grade-D oocytes in groups 1 and 2 were significantly larger than those in groups 3, 4, and 5 (p < 0.05). Only grades A and B oocytes are used for IVF and IVP procedures. The combined A+B oocyte grades did not significantly differ between groups 1 and 2, as did the combined A + B oocytes in groups 3, 4, and 5. Moreover, the number of combined grades A + B oocytes was higher in groups 3 and 4 than in groups 1 and 2, but the number of combined grades C + D oocytes was lower in groups 3 and 4 than in groups 1 and 2 (p < 0.05). Furthermore, the quantity of grades A + B oocytes was significantly greater in group 5 than in group 2, but not in groups 1, 3, or 4. Conversely, group 5 had fewer grades C + D oocytes than group 1, whereas those of groups 2, 3, and 4 did not significantly differ. Table-6 presents the average numbers of grades A–D oocytes and combined grades A + B and C + D oocytes for all groups.

## Discussion

The reduced number of high-quality oocytes obtained from OPU-IVP negatively impacts Japanese black cattle breeding. Obstacles in *in vitro* embryo production hinder the success rate of calf development from collected oocytes. The high cost of calf production result from the limited availability of top-quality oocytes through OPU. Hormonal treatment like EB before OPU could lead to a higher yield of superior oocytes compared to non-hormonal methods and be as effective as other hormonal protocols, such as GnRH + FSH + P4-CIDR + PGF_2α_, EB, and EB + CIDR + PGF_2α_. The price tag rises with heightened hormone therapies. In the beef industry, it is more cost-effective to produce more high-quality embryos using a single-dose EB treatment before OPU in the context of OPU-IVP, given the lower treatment costs.

Reports indicate that oocytes retrieved from previous hormonal treatments with OPU, specifically GnRH [12, 24, 33] and EB [2], demonstrate superior qualities compared to those without hormonal treatment. In the estrus group, cows had bigger follicles than in the diestrus group. The diestrus and estrus groups had comparable numbers of A, B, C, and D oocytes, with the highest number of D oocytes in each group. Irrespective of the phase of the estrus cycle, cows could be used for OPU without hormonal treatment, as the oocyte counts’ results were consistent. OPU oocytes collected from untreated cows showed the poorest quality. Previous reports by Fry [12], Egashira *et al*. [24], and Ogata *et al*. [33] revealed that the qualities of oocytes retrieved from previous GnRH or EB [2] treatments with OPU were superior to those without hormonal treatment with OPU.

The GnRH + FSH + CIDR + PGF_2α_ group had more medium and large follicles due to the synergistic effect of GnRH and FSH on follicle growth. Among all the EB, EB + CIDR + PGF_2α_ treatments and untreated groups, this one had the most large follicles due to the stimulation from GnRH and FSH. A previous study by Egashira *et al*. [24] showed that groups receiving GnRH and FSH therapy had comparatively larger follicles and fewer smaller follicles than other hormone-treated and untreated groups. In Japanese black cows, the gold-standard protocol for stimulating numerous large follicles for oocyte pick-up includes the use of GnRH + FSH + CIDR + PGF_2α_ [24].

In the EB and EB + CIDR + PGF_2α_ treatment groups, the cows had a larger proportion of small follicles and no large follicles compared to the other groups, while medium follicle numbers in the EB-treated groups did not significantly differ and were similar to those in the GnRH + FSH + CIDR + PGF_2α_ group. 120 h after EB treatment, Japanese black cows with EB or EB + CIDR + PGF_2α_ treatment had more medium follicles compared to the previous study by Hidaka *et al*. [2]. This occurred due to EB synchronizing the follicular wave, inducing medium follicles’ development, and subsequently suppressing large or dominant follicles [2, 34].

Although both EB treatment groups also expressed the most small follicles in each group, when OPU had more opportunity to be performed with 4.5–4.9 mm in diameter of small follicles in this study, both EB-treated groups yielded more high-quality grade-B oocytes.

Oocytes were categorized into four grades in each group based on their cumulus quality. Among the same group, the cows in the GnRH + FSH + CIDR + PGF_2α_ treatment group had the greatest number of medium and large follicles and the highest yield of grade-A oocytes for OPU. Previous research by Seneda *et al*. [35] indicates that an abundant number of dominant follicles at the time of OPU correlates with an increased COC quantity and quality. Furthermore, EB treatment of cows before OPU yields the highest number of grade-B oocytes due to the generation of the greatest number of small and medium follicles. Like a previous study by Hidaka *et al*. [2], cow ovaries demonstrated an increase in total follicles along with a larger percentage of medium follicles following EB treatment. After EB administration, the dominating follicles regressed, triggering a new follicular wave. The estrus cycle’s dominant follicles’ dynamics can be managed and synchronized using EB treatment [27]. Cows treated with EB + CIDR + PGF_2α_ produced the majority of their oocytes in grades A, B, and D while having the least amount in grade C. The authors hypothesized that the administration of P4 in CIDR form would hinder estrus and GnRH release, leading to an accumulation in the hypothalamus and subsequent stimulation of more follicle growth and enhanced oocyte quality post-withdrawal. However, comparing the same parameters between cows in the EB and EB + CIDR treatment groups showed non-significant differences in all follicle sizes and oocyte grades between the groups. P4 was administered in combination with EB to regulate and synchronize the development of many medium-sized follicles (4–6 mm in diameter) and their follicular waves, which were then selected and grew into dominant follicles while suppressing subordinate follicles [26]. However, EB treatment can also control the dynamics of dominant follicles throughout the estrus cycle and synchronize proestrus development of the dominant follicles [27]. The mean numbers of counted follicles and graded oocytes were not significantly different between the EB and EB + CIDR treatment groups.

IVP and IVF both require grades A and B oocytes [36]. Although the GnRH + FSH + CIDR + PGF_2α_ treatment groups provided the maximum number of grade A oocytes, the combined grade A + B oocyte numbers did not significantly differ between the GnRH + FSH + CIDR + PGF_2α_ and the EB and EB + CIDR + PGF_2α_ treatment groups or between the EB and EB + CIDR + PGF_2α_ treatment groups. Conversely, all hormone treatment groups except EB + CIDR + PGF_2α_ yielded more A + B oocytes than the untreated groups. Thus, the high quantity and quality of the grades A + B oocytes in crossbred Japanese black cows were equivalent to the administrations of GnRH + FSH + CIDR + PGF_2α_ versus EB and EB + CIDR + PGF_2α_ before OPU. Therefore, compared with both untreated groups, the GnRH + FSH + CIDR + PGF_2α_ and EB groups had more potential to induce more A + B oocytes [2, 24, 33], whereas the EB + CIDR + PGF_2α_ group had a strong tendency to provide more. Furthermore, the recovery rate was nearly 51% of all oocytes collected after OPU, which was lower than that recovered in previous study [1, 2, 24] by approximately 75%–80%; thus, fewer grades A + B oocytes were obtained in this study than in previous studies by Cavalieri *et al*. [1], Hidaka *et al*. [2], and Egashira *et al*. [24].

## Conclusion

The effectiveness of EB treatment in enhancing follicle growth and yielding more high-quality oocytes before OPU in donor cows is on par with GnRH + FSH + CIDR + PGF_2α_ and EB + CIDR + PGF_2α_ treatments. Using hormones was deemed advantageous and economical. In crossbred Japanese black cattle undergoing ovarian stimulation for OPU, using EB treatment with lower hormonal requirements is the most cost-effective and effective alternative protocol. Studies suggest researching phytoestrogens as a natural alternative to EB treatment for enhancing ovarian function before OPU in other beef cattle breeds.

## Authors’ Contributions

JK and AI: Participated in the experimental design and conducted the experiment. JK: Conducted a comprehensive literature search, collected and assisted in the analysis of the collected data, and drafted, revised, and proofread the manuscript. AI: Assisted in collecting and analyzing the data. Both authors have read, reviewed, and approved the final manuscript.
